# Contribution of Regulatory T Cells in Nucleotide-Binding Oligomerization Domain 2 Response to Influenza Virus Infection

**DOI:** 10.3389/fimmu.2018.00132

**Published:** 2018-01-31

**Authors:** Benoit Egarnes, Jean Gosselin

**Affiliations:** ^1^Laboratory of Innate Immunology, Centre de recherche du CHU de Québec-Université Laval, Université Laval, Quebec City, QC, Canada; ^2^Department of Molecular Medicine, Université Laval, Quebec City, QC, Canada

**Keywords:** nucleotide-binding oligomerization domain 2, muramyl dipeptide, regulatory T cells, Th17 cells, lung inflammation, influenza A virus, monocytes, neutrophils

## Abstract

Influenza A virus (IAV) is recognized to cause severe pulmonary illnesses in humans, particularly in elderly and children. One of the features associated with IAV infection is an excessive lung inflammation due to an uncontrolled immune response. The nucleotide-binding oligomerization domain 2 (NOD2) receptor is known to recognize ssRNA viruses such as IAV, but its role in the inflammatory process during viral infections remains to be clarified. In a previous report, we have shown that activation of NOD2 with muramyl dipeptide (MDP) significantly reduces both viral loads and lung inflammation and also improves pulmonary function during IAV infection. These findings prompted us to further investigate whether NOD2 receptor may contribute to regulate inflammation during viral infection. In the present study, we show that administration of MDP to mice infected with IAV stimulates the migration of regulatory T (Treg) cells to the lungs. Such a presence of Treg cells was also accompanied with a reduction of neutrophils in the lungs during IAV infection, which correlated, with a significant decrease of Th17 cells. In our model, Treg cell recruitment is dependent of CXCL12 and CCL5 chemokines. Moreover, we show that the presence of Ly6C^low^ patrolling monocytes is required for Treg cells mobilization to the lung of mice treated with MDP. In fact, following monocyte depletion by administration of clodronate liposome, mobilization of Treg cells to the lungs of treated mice was found to occur when circulating Ly6C^low^ monocytes begin to reemerge. In addition, we also detected an increased production of TGF-β, a cytokine contributing to Treg activity when blood Ly6C^low^ monocytes are restored. Together, our results demonstrate that MDP treatment can promote an anti-inflammatory environment through the mobilization of Treg cells to the lung, a mechanism that requires the presence of Ly6C^low^ monocytes during IAV infection. Overall, our results suggest that activation of NOD2 receptor could be an appealing approach to control pulmonary inflammation in patients infected with IAV.

## Introduction

Influenza virus is responsible for annual epidemic infection around the world causing severe morbidity, mostly among elderly, children, and people with chronic pulmonary disease. Influenza virus has the capacity to interact with various cell populations in the lungs that contribute to the earliest wave of production of type 1 interferon and inflammatory cytokines which in turn will trigger local and systemic responses against the virus. When not well controlled, virally infected cells can be extensively targeted by innate cells that may result in an excessive production of inflammatory mediators and an enhanced pulmonary inflammation and lung illness (1–3).

Regulatory T cells (Treg) are a subpopulation of CD4^+^ T cells that have been identified as suppressors of immune responses and inflammation (4). Tregs are recognized as CD4^+^ CD25^+^ T cells which specifically express the transcription factor FoxP3 (5, 6). They play a central role in the maintenance of immunological tolerance and are also known to maintain self-tolerance and prevent autoimmune and chronic inflammatory diseases (7–9). Suppressive functions of Treg cells are known to be associated with TGF-β inhibitory effects and critical for immune homeostasis (10, 11). The role of TGF-β in controlling T cells functions and immune responses has been extensively studied by acting as a key regulator of the signaling pathway that promotes the generation of Tregs from CD4^+^ CD25^−^ precursors (12). It was proposed that Tregs can control immune balance during viral infection and to limit the extent of tissue damage that occurs in the course of infection (13–15). For example, Tregs appear essential to clear influenza virus infection in neonatal mice (16), as their depletion results in enhanced lung inflammatory response to IAV infection. In line with these observations, it was also demonstrated that Tregs contribute to the resolution of lung inflammation after influenza virus infection (17). Conversely, other studies reported that Tregs have little effects on severity of disease after infection (18, 19). In fact, it was shown that administration of Treg neutralizing antibodies to infected mice has no significant effects on body weight loss, mortality, and viral clearance, suggesting that this cell population has a limited role in controlling IAV infection.

Early excessive neutrophil infiltration to the lung is a well-characterized symptom of IAV infection (20–22). Th17 cells through IL-17A production can mobilize neutrophils to the site of inflammation (23, 24). A prolonged presence of Th17 and neutrophils could thus impair tissue integrity. Since the balance between Tregs and Th17 cells is essential for homeostasis, a loss of this delicate balance may result in deleterious effect on an efficient control of inflammation during IAV infection (25–27).

Recognition of pathogens by host innate sensors is a prerequisite for the initiation of immune response. While initially reported to recognize bacterial component such as bacterial peptidoglycan-derived muramyl dipeptide (MDP) (28, 29), nucleotide-binding oligomerization domain 2 (NOD2), a member of the nucleotide-binding oligomerization domain-like receptors, also has the capacity to bind ssRNA from various viruses including influenza A virus (IAV) (30). Importance of this receptor in controlling influenza infection was supported in *Nod2*^−/−^ mice, which were found to be hypersusceptible to infection, and showed enhanced pulmonary airway obstruction (30, 31). Previous work from our laboratory has demonstrated that treatment of influenza virus-infected mice with MDP induces the production of type I interferon, reduces lung viral loads and inflammation, and improves pulmonary function (32). While the mechanisms associated with NOD2 in the control of inflammation remain to be identified, a previous study has suggested a functional relevance of NOD2 in Treg biology (33). Indeed, polymorphism of NOD2 receptor that characterizes patients with Crohn’s disease has been associated with a deficiency in Treg cells levels.

In the present study, we demonstrate that treatment of IAV-infected mice with NOD2 agonist, MDP, significantly increases Treg cells mobilization to the lungs. Such presence of Tregs in lungs of infected mice correlates with a reduced number of Th17 cells and infiltrated neutrophils. Furthermore, functional depletion of peripheral blood monocytes with clodronate liposome administration, results in an impaired mobilization of Treg cells to the lungs, suggesting the essential role of monocytes, and predominantly Ly6C^low^ monocytes in this process. Together, our results shed light on innate mechanisms involved in the NOD2-mediated regulation of lung inflammation in the course of IAV infection.

## Materials and Methods

### Ethics Statement

This study was carried out in accordance with the recommendations of the Guide for the Care and Use of Laboratory Animals of the Canadian Council on Animal Care. All protocols were approved by the Committee on the Ethics of Animal Experiments of Université Laval (Approval Number: 15-109-2).

### Mice

The 4- to 6-week-old C57Bl/6 wild-type (WT) mice were obtained from Charles River (St-Constant, QC, Canada) and NOD2-deficient mice (*Nod2^−/−^*) from Jackson Laboratory. All mouse colonies and littermates were housed in a controlled environment and a specific pathogen-free animal facility at the Centre de Recherche du CHU de Quebec, Laval University.

### Viral Infections

Infections were achieved using Influenza virus (IAV) strain A/Puerto Rico/8/34 (H1N1). IAV was disseminated and extracted from Madin-Darby canine kidney (MDCK) cells. MDCK cells were titrated using standard plaque assay as reported (34). Animals were infected intranasally (i.n.), at day 0 of the protocols, with a sublethal dose of IAV [50 plaque forming unit (PFU)]. We daily assessed the general health of the animal by monitoring their physical appearance, body weight and temperature.

### *In Vivo* Treatments

MDP (Invivogen) was diluted in saline (0.9% p/v) and intravenously (i.v.) injected in the tail vein of mice at 10 mg/kg. Treatments started one day after IAV-infection. Control mice were injected with saline (0.9% p/v) (placebo). Mice were treated daily and were sacrificed at indicated times.

### Depletion of Blood Monocytes

Mouse blood monocytes were depleted using dichloromethylene-biphosphonate (clodronate)-loaded liposomes (Clodronate liposomes, Amsterdam, Netherlands) as previously described (35, 36). Clodronate-loaded liposomes (200 µl) were injected in mice tail vein, 24 h prior to influenza virus infection, unless otherwise indicated. Control animals received PBS-loaded liposomes. Monocytes depletion efficiency was monitored at indicated times by flow cytometry.

### Flow Cytometry Analysis

Single-cell suspensions obtained from blood or collagenase and DNase-digested lungs were first incubated with anti-CD16/32 (clone 93 BioLegend, San Diego, CA, USA) to block non-specific antibody interaction with Fc receptors. Fixable viability dye eFluor^®^450 (eBioscience, San Diego, CA, USA) was used to identify live cells following cellular fixation/permeabilization. For intracellular cytokine staining, cells were first stimulated for 5 h at 37°C with 50 ng/ml PMA, 1 µg/ml ionomycin (Sigma-Aldrich, St. Louis, MO, USA) and with 10 µg/ml of GolgiStop (BD Biosciences, San Diego, CA, USA). Tregs cells were identified using anti-CD4 (clone RM4-5; BioLegend San Diego, CA, USA), anti-CD25 (clone PC61; BioLegend San Diego, CA, USA), and anti-FoxP3 (clone FJK-16s; eBioscience, San Diego, CA, USA). Th17 cells were identified using IL-17A (clone eBio17B7 eBioscience, San Diego, CA, USA), and anti-CD4. Blood monocytes and neutrophils were identified using anti-CD45 (clone 30F11; BD Biosciences, San Diego, CA, USA), anti-CD115 (clone AFS98; BioLegend, San Diego, CA, USA), anti-Ly6G (clone 1A8; BD Biosciences), anti-CD11b (clone M1/70; BD Biosciences), and anti-Ly6C (clone HK1.4; BioLegend, San Diego, CA, USA). Flow cytometry was performed using BD LSR II (BD Biosciences, ON, Canada) and data analyzed with FACSDiva software Version 6.1.2 (BD Biosciences, ON, Canada). Absolute count numbers for cell populations were calculated using the BD Trucount™ tubes (BD Bioscience) according to manufacturer’s instructions.

### Cytokines Measurement in Lungs Homogenates

Levels of TGF-β1 and CXCL12 (R&D Systems, Minneapolis, MN, USA) were determined by ELISA. Levels of IL-10, IL-17A, CCL5, TNFα, IL-6, and KC were determined using BD Cytometric Bead Array system (CBA Flex Set; BD Biosciences). Samples were analyzed with a BD FACS CANTO II flow cytometer and cytokine concentrations were evaluated with FCAP Array software (BD Biosciences). Results are expressed in pg/ml of lung homogenates.

### CXCL12 and CCL5 Neutralization

CXCL12 (SDF-1) was neutralized using anti-SDF-1 (clone K15C; Millipore, Massachussetts, NE, USA) antibody as previously described (37) and CCL5 was neutralized using anti-CCL5 (PeproTech, Rocky Hill, NJ, USA) antibody as described elsewhere (38). Neutralizing antibodies (32 μg/mouse) or IgG2 control isotype (32 μg/mouse) were intraperitoneally (i.p.) injected 24 h prior to IAV infection in WT mice. Thereafter, mice were treated daily with MDP and sacrificed at day 5 postinfection.

### Immunofluorescence Analysis

Lungs sections were fixed in paraformaldehyde for 15 min and then washed with PBS (3 × 15 min). Sections were incubated at room temperature in blocking solution containing PBS, 0.4% Triton X-100, 4% rat serum, and 0.5% bovine serum albumin for 20 min. Sections were then rinsed with PBS and stained overnight at 4°C with Ly6C-FITC (clone ER-MP 20; Abcam, Cambridge, UK) and Ly6G-A594 (clone 1A8; Biolegend, San Diego, CA, USA) antibodies. After extensive wash in PBS, lung sections were incubated with Hoechst 33342 for 15 min and mounted in Fluoromount medium for visualization under a confocal spinning disk microscope (Quorum wave FX, Quorum Technologies, Ontario, Canada).

### Histological Analysis

Wild-type and *Nod2*^−/−^ mice were infected with IAV (50 PFU) and daily treated with placebo (0.9% p/v saline) or MDP (10 mg/kg). Lungs were harvested at day 5 postinfection and fixed in PFA (4%). Tissues were embedded in paraffin and lungs sections were stained with hematoxylin and eosin for histological analysis (39).

### Statistical Analysis

All analyses were performed using Graph Pad Prism version 6.02 software (Graph Pad Software, San Diego, CA, USA). Statistical significance was set at *p* < 0.05. Differences in groups were determined with a two-way analysis of variance (ANOVA) followed by a Tukey *post hoc* test or otherwise indicated.

## Results

### MDP Treatment Increases Levels of Treg Cells in Lungs of IAV-Infected Mice

**Figure 1:**
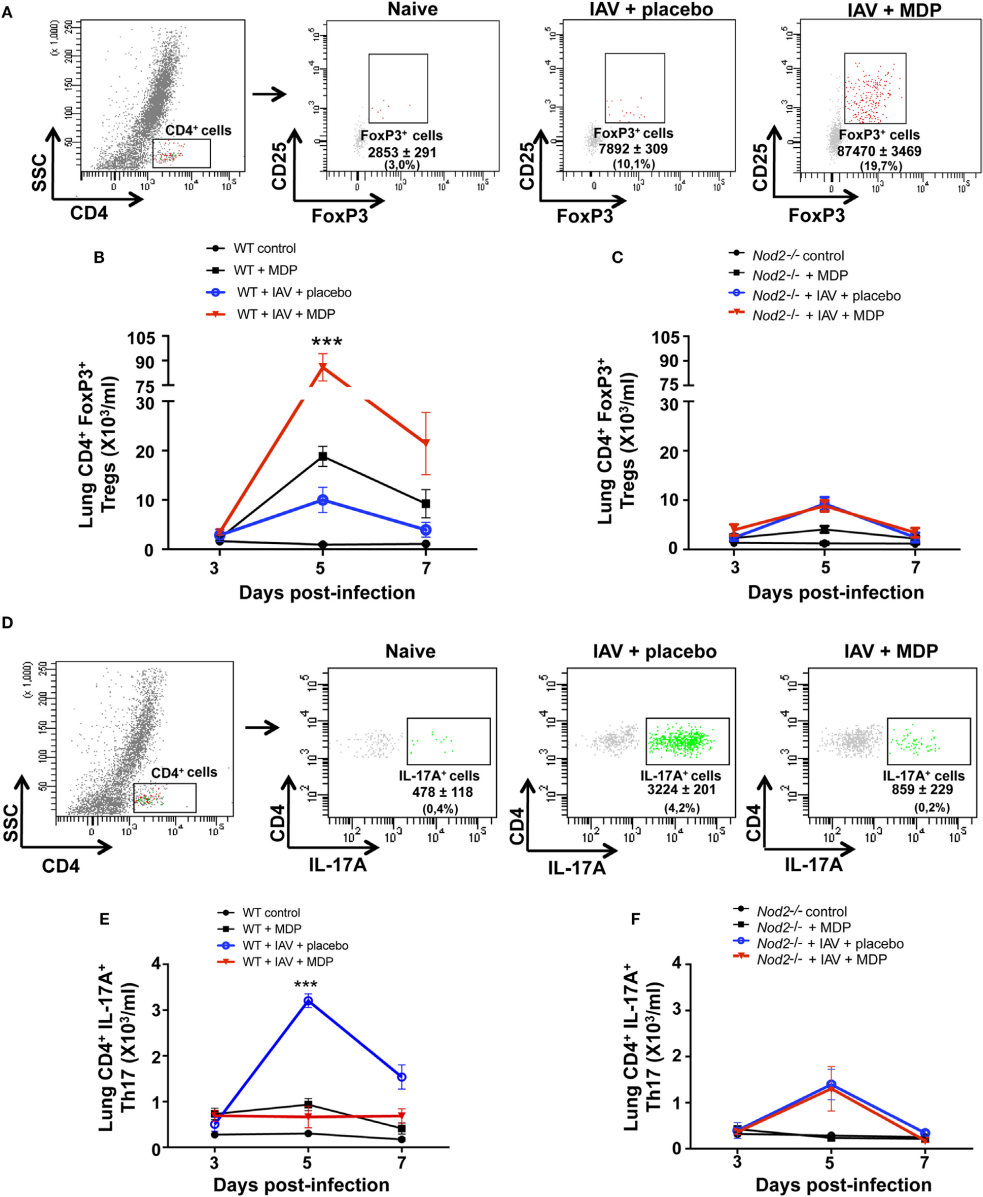
Muramyl dipeptide (MDP) treatment increases levels of regulatory T (Treg) cells in lungs of influenza A virus (IAV)-infected mice. **(A)** Representative gating strategy of Treg cells (CD4^+^ CD25^+^ FoxP3^+^) in lungs of naive and IAV-infected wild-type (WT) mice at day 5 postinfection. Mice were daily treated with placebo or MDP. Numbers in parentheses express the percentage (%) of Treg cells on CD4^+^ T population. Number of Treg cells in lungs of **(B)** WT and **(C)** *Nod2*^−/−^ mice infected with IAV and daily treated with placebo or MDP as measured by flow cytometry. Lungs samples were collected at days 3, 5, and 7 postinfection. **(D)** Representative gating strategy of Th17 cells (CD4^+^ IL-17A^+^) in lungs of naive and IAV-infected WT mice at day 5 postinfection. Numbers in parentheses express the percentage (%) of Th17 cells on CD4^+^ T population. Number of Th17 cells in lungs of **(E)** WT and **(F)** *Nod2^−/−^* mice infected with IAV and daily treated with placebo or MDP as measured by flow cytometry. Lungs samples were collected at indicated time post-infection. Results are presented as mean ± SEM of two independent experiments (*n* = 5 mice/groups). Differences in groups were determined with two-way analysis of variance followed by a Tukey *post hoc* test. ***p < 0.001, IAV-infected mice treated with placebo as compared to IAV-infected animals treated with MDP.

Excessive inflammation and structural damages to the lungs are consequences of infection by influenza virus (IAV) (40). Previous studies suggest that Treg cells can play a central role in immune homeostasis during viral infection (13, 16). Since we previously reported that administration of MDP to mice infected with IAV reduces lung inflammation (32), we first wanted to determine if such effects of MDP treatment could lead to the mobilization of Treg cells in lungs of IAV-infected mice. WT and NOD2-deficient mice (*Nod2^−/−^*) were infected with IAV and treated daily with MDP or placebo. At days 3, 5, and 7 postinfection (p.i.), Treg (CD4^+^ CD25^+^ FoxP3^+^) cells were assessed in lungs of IAV-infected mice. We observed in mice treated with MDP that number of Treg cells significantly increase at day 5 p.i. in lungs of IAV-infected animals to decline thereafter (Figures 1A,B). Similarly, lung viral loads were significantly reduced after 5 days of treatment with MDP (data not shown). Such effects of MDP treatment on Treg cells mobilization were strongly prevented in *Nod2*^−/−^ mice in lungs (Figure 1C). Comparable results were obtained when Treg frequencies were measured in blood of mice infected with IAV (data not shown).

Mobilization and activation of Treg cells in the inflamed organ or tissue are closely related to the generation of Th17 cells. We have thus determined whether MDP treatment may affect this cell population during IAV infection. As expected, we observed that IAV infection induce the recruitment of Th17 cells into the lungs of mice. In contrast, in mice treated with MDP, a significant decrease of Th17 cell mobilization was observed in lungs of IAV-infected WT mice (Figures 1D,E). Again, effects of MDP on Th17 were abolished in *Nod2*^−/−^ mice (Figure 1F).

**Figure 2:**
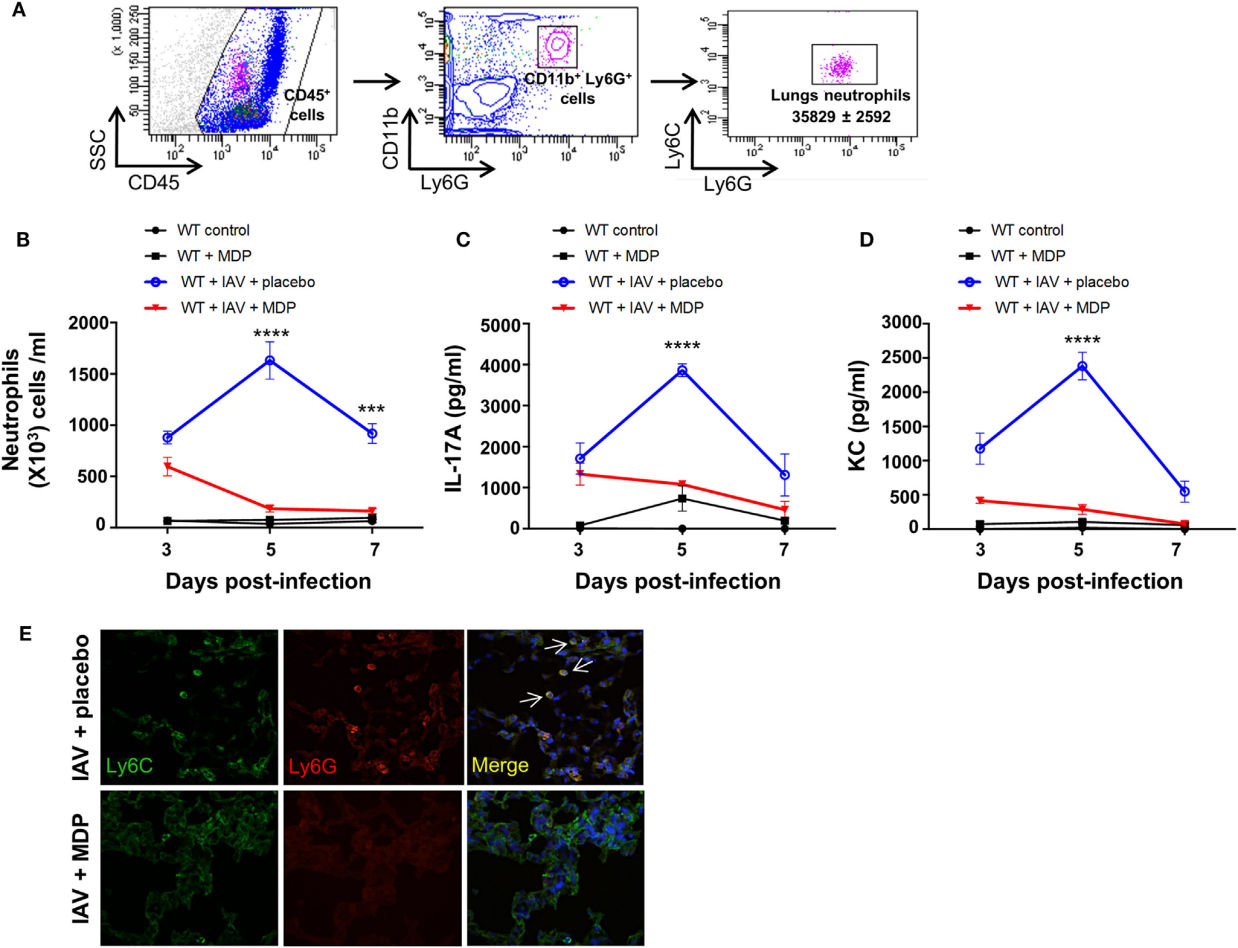
Muramyl dipeptide (MDP) treatment reduces neutrophils migration to the lungs of influenza A virus (IAV)-infected mice. **(A)** Representative gating strategy of lungs neutrophils (CD45^+^, CD11b^+^, Ly6G^+^, Ly6C^+^) in wild-type (WT) naive mice. **(B)** Number of neutrophils was measured in lung tissues of IAV-infected mice treated with MDP or placebo at indicated time post-infection. Levels of **(C)** IL-17A and **(D)** KC were determined in lung homogenates of IAV-infected WT mice daily treated with placebo or MDP. Lungs were collected at days 3, 5, and 7 postinfection. Results are presented as mean ± SEM of two independent experiments (*n* = 4 mice/groups). Differences in groups were determined with two-way analysis of variance followed by a Tukey *post hoc* test. ***p < 0.001 and ****p < 0.0001, as compared to IAV-MDP-treated mice **(E)** Immunofluorescence analysis of lungs sections from IAV-infected mice daily treated with placebo or MDP. Lungs were extracted at day 5 postinfection and lung sections were stained with Hoechst (blue), Ly6C (green), and Ly6G (red) antibodies. Arrows show Ly6C^+^ and Ly6G^+^ neutrophils. Images are representative of one experiment (*n* = 4 mice/group).

Excessive secretion of interleukin-17A (IL-17A) by Th17 cells may sustain neutrophil migration to the lungs following influenza infection (20–22), and therefore contribute to disease in severe influenza infection (41–43). Since our results show that administration of MDP leads to a decrease of Th17 cells mobilization to lungs of IAV-infected mice, we next wanted to determine whether neutrophils recruitment is also modulated by MDP treatment. WT mice were infected with IAV and treated daily with MDP or placebo. At days 3, 5, and 7 post-IAV infection, we assessed the levels of neutrophils in lung of mice (Figure 2A) as well as the concentration of IL-17A and KC, two mediators involved in neutrophil recruitment (44–46). IAV infection induces maximal recruitment of neutrophils in lungs of mice at 5 days postinfection, which correlates with increase of IL-17A and KC production (Figures 2B–D). In contrast, MDP treatment induces a significant decrease of neutrophils, IL-17A and KC production in lungs of IAV-infected animals at the same time point compared to the placebo groups. These results are supported by immunofluorescence analysis showing that MDP treatment decreases neutrophils (Ly6G^+^, Ly6C^+^) infiltration to the lungs of IAV-infected mice (Figure 2E). These first sets of experiments demonstrate that MDP treatment could induce recruitment of Treg cells to the lungs of IAV-infected mice but also favors the balance between Treg and Th17 cells in lungs of infected animals.

### CXCL12 and CCL5 Contribute to Treg Cell Trafficking following MDP Treatment

**Figure 3:**
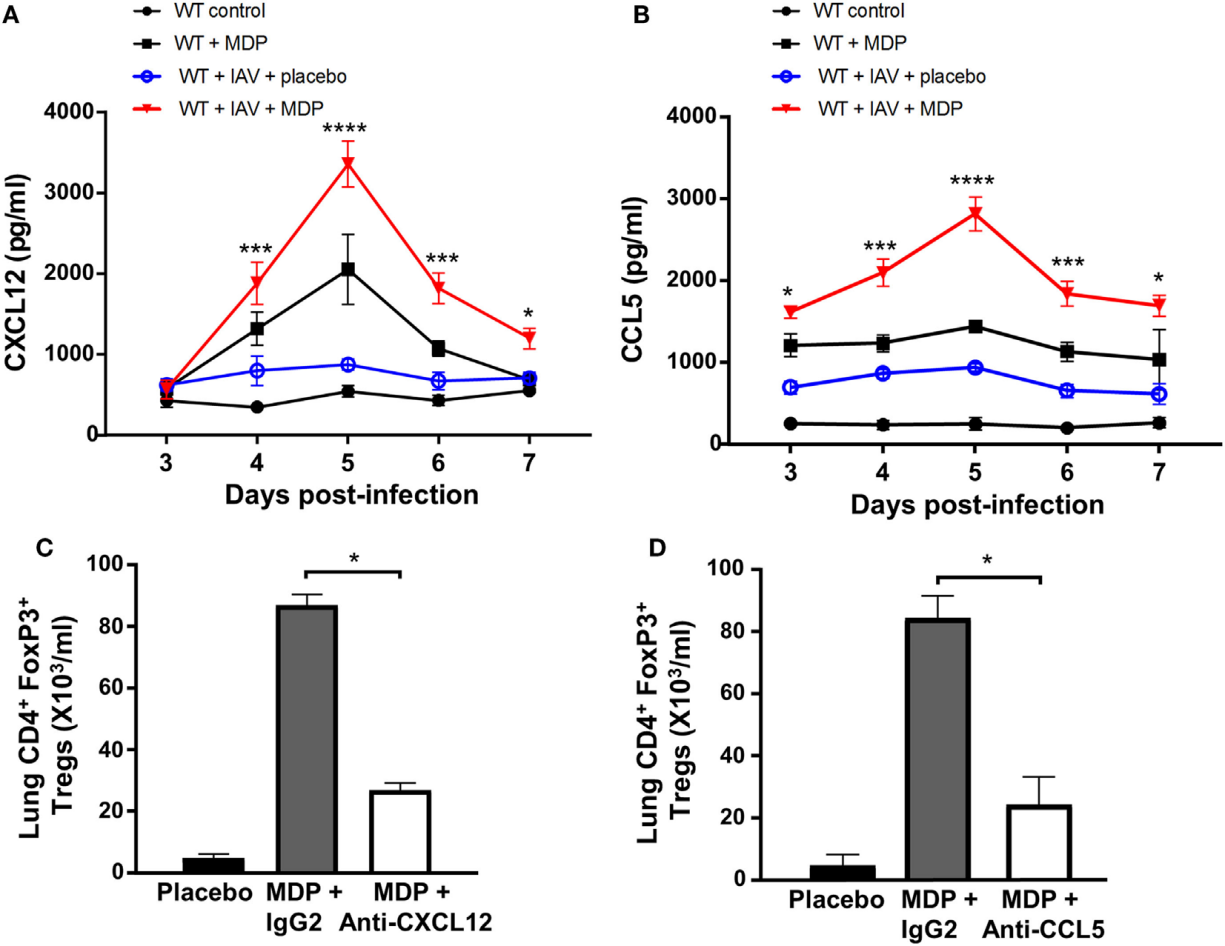
CXCL12 and CCL5 contribute to regulatory T (Treg) cell trafficking following muramyl dipeptide (MDP) treatment. Levels of **(A)** CXCL12 and **(B)** CCL5 production were measured in lung homogenates of influenza A virus (IAV)-infected wild-type (WT) mice daily treated with placebo or MDP. Lungs were collected at days 3, 4, 5, 6, and 7 postinfection. Results are presented as mean ± SEM of two independent experiments (*n* = 4 mice/groups). Differences in groups were determined with a two-way analysis of variance followed by a Tukey *post hoc* test. *p < 0.05, ***p < 0.001, and ****p < 0.0001 as compared to IAV-infected mice treated with placebo. **(C,D)** Number of Treg cells to the lungs was analyzed by flow cytometry in IAV-infected mice injected with **(C)** anti-CXCL12 or **(D)** anti-CCL5 neutralizing antibodies and daily treated with MDP or placebo. Neutralizing antibodies or IgG2 isotype control (32 μg/mice i.p.) was administered one day prior to IAV infection. Lungs were collected at day 5 postinfection. Results are presented as mean ± SEM of two independent experiments (*n* = 3 mice/groups). Differences in groups were determined with a Mann–Whitney test *p < 0.05 when compared with indicated groups.

Various chemokine receptors and integrins are involved in Treg cell trafficking. The CXCR4 receptor and its ligand CXCL12 appear to be crucial for bone marrow trafficking (47). CCR5 receptor was also found to be essential for recruitment of Treg cells to lymphoid tissues and organs including lungs (48). Since we observed that administration of MDP leads to the recruitment of Treg cells to the lungs of infected mice, we next wanted to determine whether CCL5, also known as RANTES, and CXCL12 chemokines may contribute to Treg cell mobilization after MDP treatment. We observed a marked increase of both CXCL12 and CCL5 at day 5 p.i. in lungs of mice following treatment with MDP compared to the control groups (Figures 3A,B). Importance of these chemokines in the mobilization of Treg cells to the lungs was demonstrated by the administration of anti-CXCL12 and anti-CCL5 neutralizing antibodies to mice prior infection and treatment with MDP. Indeed, numbers of recruited Tregs were found to significantly decrease by at least 60% in lungs of mice receiving neutralizing antibodies (Figures 3C,D). These results highlight the role of both CCL5 and CXCL12 in Treg cells migration induced by MDP.

### MDP Treatment Increases TGF-β and IL-10 Secretion in Lungs of IAV-Infected Mice

**Figure 4:**
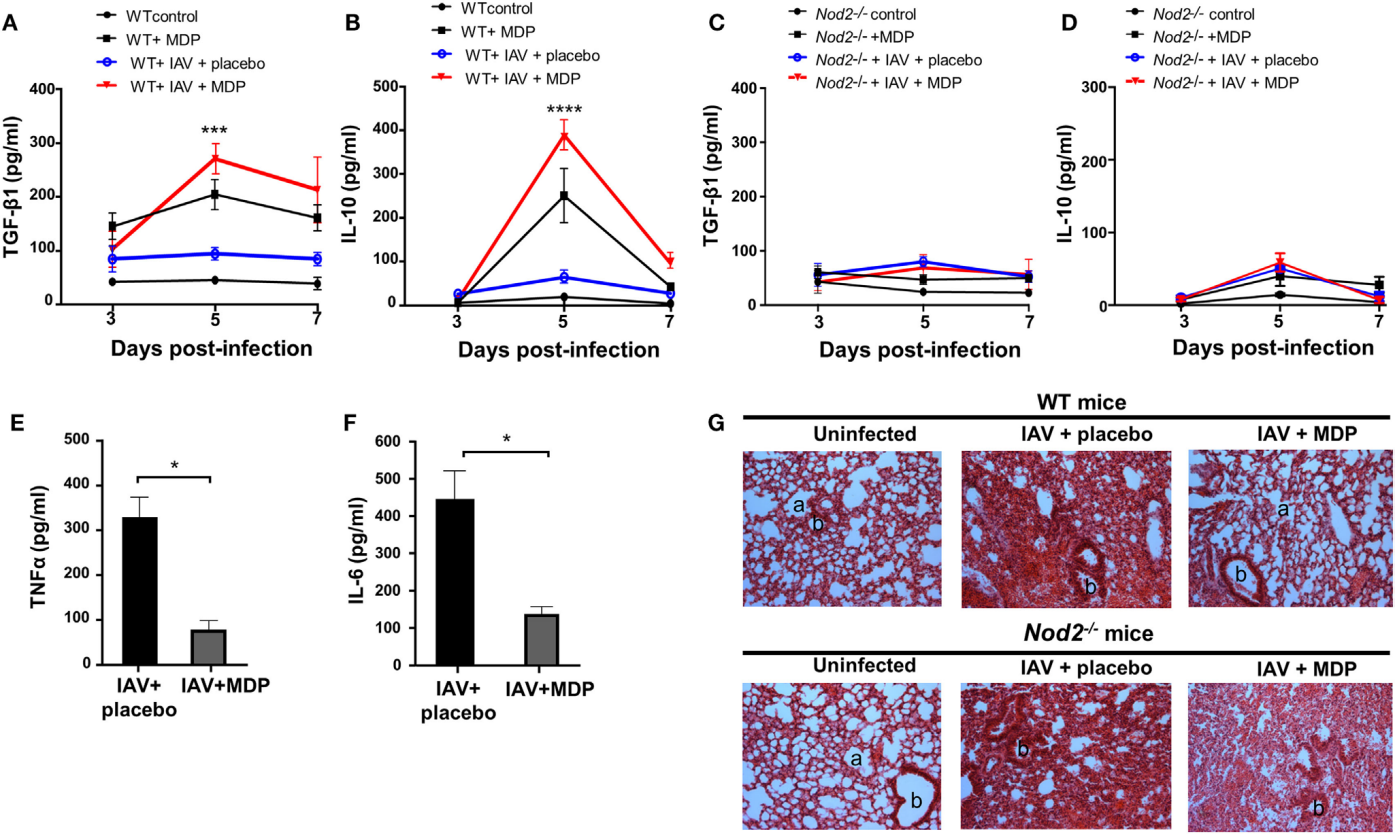
Muramyl dipeptide (MDP) treatment increases TGF-β and IL-10 secretion in lungs of influenza A virus (IAV)-infected mice. Levels of **(A,C)** TGF-β1 and **(B,D)** IL-10 were measured in lungs homogenates of IAV-infected wild-type (WT) (left panel) and *Nod2^−/−^* (right panels) mice daily treated with placebo or MDP. Lungs were collected at days 3, 5, and 7 postinfection. Results are presented as mean ± SEM of two independent experiments (*n* = 4 mice/groups). Differences in groups were determined with a two-way analysis of variance followed by a Tukey *post hoc* test. ***p < 0.001 and ****p < 0.0001 as compared to IAV-infected mice treated with placebo. Levels of **(E)** TNFα and **(F)** IL-6 were assessed in lung homogenates of IAV-infected WT mice daily treated with placebo or MDP. Lungs were harvested at day 5 postinfection. Results are presented as mean ± SEM of two independent experiments (*n* = 4 mice/groups). Differences in groups were determined with a Mann–Whitney test. *p < 0.05 when compared with indicated groups. **(G)** Hematoxylin and eosin-stained lung sections from uninfected or IAV-infected WT and *Nod2*^−/−^ mice, treated daily with placebo or MDP. Lungs were harvested at day 5 postinfection. Images are representative of two independent experiments (*n* = 3 mice/group). a: example of alveolar and b: bronchiolar structure (original magnification 100×).

The importance of TGF-β and IL-10 in Treg-mediated suppression was demonstrated in several *in vivo* models (49, 50). Next, we determined whether the mobilization of Treg cells to the lung of infected mice treated with MDP correlates with the production of TGF-β and IL-10. Treatment with MDP leads to a gradual increase of TGF-β secretion in lungs of IAV-infected mice which was most pronounced at day 5 p.i. compared to the control groups. Production of IL-10 was mainly detectable at the same time point but rapidly decreased thereafter (Figures 4A,B). Similar results were obtained in sera of infected mice treated with MDP (data not shown). As expected, such effects of MDP treatment on TGF-β and IL-10 synthesis were abrogated in mice deficient for NOD2 receptor (Figures 4C,D).

In line with these observations, treatment with MDP reduces production of inflammatory cytokines such as TNFα and IL-6 in lungs of infected mice (Figures 4E,F). Treatment with MDP also induced histological changes in lungs of infected mice which is characterized by a reduced interstitial leukocyte infiltrates (Figure 4G). These results suggest that MDP treatment may control excessive lung inflammation by regulating the production of TGF-β and IL-10 in mice infected with IAV.

### Ly6C^low^ Blood Monocytes Contribute to Treg Cell Mobilization into the Lungs of IAV-Infected Mice Treated with MDP

**Figure 5:**
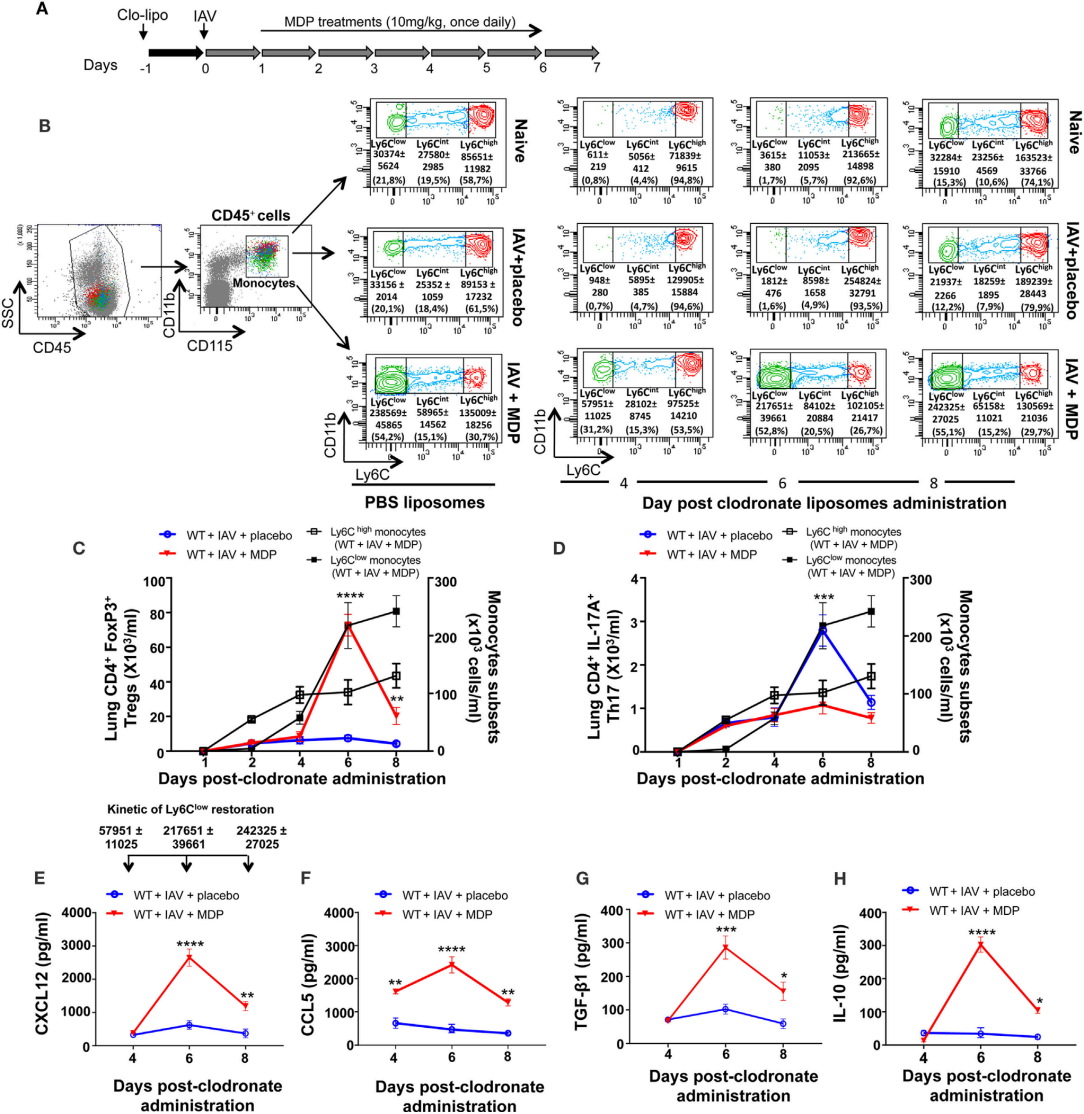
Ly6C^low^ monocytes are involved in regulatory T (Treg) cells mobilization to lungs of influenza A virus (IAV)-infected mice. **(A)** Experimental design of blood monocyte depletion. Briefly, 1 day prior to IAV infection, mice were treated with clodronoate-liposome (200 µl i.v.) to deplete blood monocytes. **(B)** Gating strategy of blood monocytes subsets. After gating out the neutrophil cell population (CD45^+^/CD11b^+^/Ly6G^+^), monocytes were identified as CD45^+^, CD11b^+^, and CD115^+^. Monocyte subsets were further subdivided in three populations based on their expression level of Ly6C: Ly6C^high^, Ly6C^int^, and Ly6C^low^. Flow cytometry analysis of blood monocytes subsets was assessed at day 6 following PBS liposome administration and at days 4, 6, and 8 postclodronate liposome injection. **(C)** Treg and **(D)** Th17 cells numbers in lung tissues were measured by flow cytometry at days 1, 2, 4, 6, and 8 postclodronate administration in IAV-infected mice daily treated with placebo or muramyl dipeptide (MDP). Number of Ly6C^high^ and Ly6C^low^ monocytes (black curves) in the blood of IAV-infected mice treated with MDP is shown to illustrate monocytes subsets replenishment following clodronate liposome administration. Results are presented as mean ± SEM of two independent experiments (*n* = 4 mice/groups). Differences in groups were determined with a two-way analysis of variance followed by a Sidak *post hoc* test. **p < 0.01, ***p < 0.001, and ****p < 0.0001, IAV-infected mice treated with placebo (blue) compared to IAV-infected MDP-treated animals (red). Levels of CXCL12 **(E)**, CCL5 **(F)**, TGF-β1 **(G)**, and IL-10 **(H)** in lung homogenates were determined at days 4, 6, and 8 postclodronate administration in mice infected with IAV and daily treated with placebo or MDP. Arrows [in panel **(E)**] show the number of restored Ly6C^low^ monocytes in the blood of WT IAV-infected mice treated with MDP at days 4, 6, and 8 postclodronate liposome administration. This kinetic of Ly6C^low^ monocytes restoration is applicable to figure **(E–H)**. Results are presented as mean ± SEM of two independent experiments (*n* = 4 mice/groups). *p < 0.05, **p < 0.01, ***p < 0.001, and ****p < 0.0001, when compared with IAV-infected mice treated with placebo.

We have previously reported that Ly6C^low^ patrolling monocytes are important for trafficking of Treg cells in a mouse model of arthritis and that *in vivo* administration of MDP increases levels of Ly6C^low^ monocytes in the blood of mice (51, 52). We wanted to determine whether Ly6C^low^ monocytes are essential to Treg recruitment in lung of mice during IAV infection. To achieve this goal, we have performed functional depletion of blood monocytes subsets using intravenous injection of clodronate-liposome. This approach has no effects on the number of alveolar macrophages. Kinetic of monocyte depletion experiment is presented in Figure 5A. In naive and IAV-infected mice treated with placebo, we observed that Ly6C^low^ patrolling monocytes gradually remerge at day 6 postclodronate and are fully restored by day 8 (Figure 5B). However, a more rapid emergence of Ly6C^low^ patrolling monocytes was observed following MDP treatment, starting to re-emerged at day 4 postclodronate administration due to the conversion of Ly6C^high^ into Ly6C^low^ monocytes following NOD2 triggering (52).

In parallel, we assessed levels of Treg and Th17 cells mobilization to the lungs of IAV-infected mice at different times after administration of clodronate. We found that the number of Treg cells in mice treated with MDP transiently increase in lungs of infected animals after 6 days, when circulating Ly6C^low^ monocytes are fully restored (Figure 5C). Absence of Ly6C^low^ monocytes did not affect trafficking of Th17 cells to the lungs of IAV-infected mice treated with MDP (Figure 5D). In line with these data, optimal secretion of CXCL12 and CCL5 chemokines and of the suppressive TGF-β and IL-10 was also measured at day 6 postclodronate administration when Ly6C^low^ monocytes are present in the circulation (Figures 5E–H). These results suggest that MDP-induced Treg cell mobilization to the lung requires the presence of blood Ly6C^low^ monocytes.

**Figure 6:**
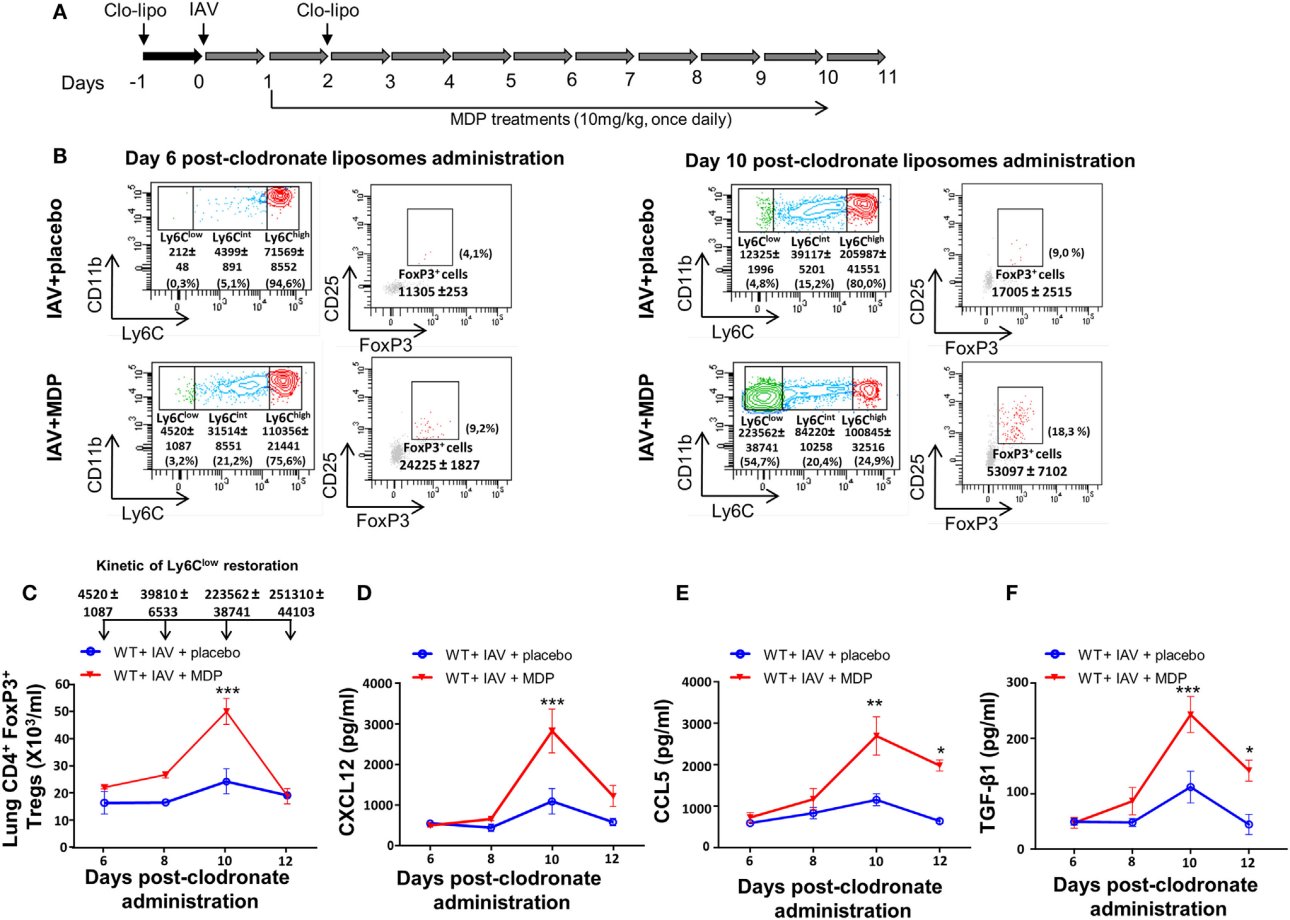
Sustained depletion of Ly6C^low^ monocytes delays muramyl dipeptide (MDP)-induced regulatory T (Treg) recruitment in lungs of influenza A virus (IAV)-infected mice. **(A)** Experimental design of blood monocyte depletion. **(B)** Monocytes subsets quantification was measured at days 6 (left panels) and 10 (left panels) postclodronate liposome administration in IAV-infected mice daily treated with placebo or MDP. Flow cytometry analysis show monocyte subset replenishment at indicated times. In parallel, Treg cells renewal is also monitored at days 6 (right panels) and 10 (right panels) postclodronate liposome administration in IAV-infected mice daily treated with placebo or MDP. Numbers in parentheses express the percentage (%) of Treg cells on CD4^+^ T population. **(C)** Absolute numbers of lungs Tregs cells were measured at indicated time following clodronate liposome administration. Arrows show the number of blood Ly6C^low^ monocytes detected in wild-type IAV-infected mice treated with MDP at days 6, 8, 10, and 12 postclodronate liposome administration. This kinetic of Ly6C^low^ monocytes restoration applies to figure **(C–F)**. Levels of **(D)** CXCL12, **(E)** CCL5, and **(F)** TGF-β1 in lung homogenates were measured at days 6, 8, 10, and 12 postclodronate administration in mice infected with IAV and daily treated with placebo or MDP. Results are presented as mean ± SEM of two independent experiments (*n* = 4 mice/groups). Differences in groups were determined with a two-way analysis of variance followed by a Sidak *post hoc* test. *p < 0.05, **p < 0.01, and ***p < 0.001, when compared with IAV-infected animals treated with placebo.

To further confirm the contribution of Ly6C^low^ patrolling monocytes in mobilization of Tregs in lungs of infected mice treated with MDP, we delayed the replenishment of blood monocyte subsets by two consecutive administrations of clodronate-liposome as detailed in Figure 6A. In this case, patrolling monocytes only started reemerging in blood by day 10 postclodronate injection. We hypothesized that if we delay the renewal kinetic of Ly6C^low^ monocytes, the recruitment of Tregs to the lungs of mice should also be affected by the absence of this monocyte subset. In IAV-infected mice treated with a placebo, we confirmed that Tregs were detected by day 10 after clodronate administration when Ly6C^low^ monocytes begin to reemerge. We also confirmed that treatment of infected mice with MDP markedly increases the presence of Tregs to the lungs of mice (Figures 6B,C). This is consistent with our previous report showing that MDP treatment induces the conversion of blood Ly6C^high^ monocytes into Ly6C^low^ monocytes and consequently their number in the circulation (52). Presence of Tregs in lungs of mice also coincides with the optimal production of CXCL12, CCL5 and TGF-β in lungs of IAV-infected mice treated with MDP compared to mice treated with a placebo (Figures 6D–F). Thus, these results indicate that the presence of Ly6C^low^ monocytes is essential to MDP to stimulate Treg recruitment to the lungs of mice.

## Discussion

While NOD2 receptor is recognized as a natural sensor of microbial fragments, its role in the regulation of inflammatory response remain to be explored. In a previous work, we have shown that NOD2 triggering by MDP treatment improves mice pulmonary function during IAV infection, suggesting that NOD2 pathway may contribute to regulate lung inflammation (32). In the present study, we demonstrate that Treg cells are associated with NOD2-mediated signals that participate to regulate lung inflammation in IAV-infected mice.

We found that triggering of NOD2 can provide physiological signals leading to the emergence of Treg cells. However, although the mechanism(s) that control such an emergence of Treg cells remain to be solved, we believe that secretion of TGF-β induced after NOD2 triggering plays a significant role in this process. It is known that TGF-β is a critical factor regulating Treg cell development by converting naive CD4^+^ T cells into CD4^+^ CD25^+^ T cells in the periphery and by inducing expression of FoxP3 in these cells (12, 53–56). Our results show that MDP treatment significantly increases Treg cells mobilization in lungs of IAV-infected mice which coincide with an increased production of TGF-β, a cytokine intimately associated with Treg function. Although various cell populations can produce TGF-β, we believe that Ly6C^low^ monocytes are a major source of TGF-β, as we have previously proposed (51). The reduction of inflammatory mediators such as TNFα and IL-6 along with an increase of both TGF-β production and Treg cell levels in lungs of mice treated with MDP are also consistent with an improved tissue integrity. We also observed that emergence of CD4^+^ Tregs in lungs of infected mice treated with MDP is transient, e.g., their numbers rapidly decrease when lung viral loads are reduced after treatment and when tissue integrity is restored. Such a kinetic of Treg trafficking was seen in other models of inflammation (57, 58). It is thus plausible that the observed rapid decrease of Treg cells could be related to the duration of the infection. Indeed, it appears likely that CD4^+^ Tregs could play a more consistent effect in response to chronic or persistent infection in which ample time is available for Treg activation and mobilization (13, 59, 60). An optimal production of IL-10 in lungs of IAV-infected mice treated with MDP was also found to coincide with the kinetic of Treg cell mobilization and the resolution of phase of infection. While various immune effector cells can release IL-10, this mediator can be markedly produced by CD4^+^ Treg cells following MDP treatment given the known connection between CD4^+^ Treg cells and IL-10 in the suppression of Th17 cells and in promoting the formation of CD8^+^ T cells during viral infection (55, 61–63). In our model, we believe that CD4^+^ Treg cells could play a central role in protecting host following NOD2 triggering by MDP, a process requiring the production of TGF-β. Although we demonstrated that activation of NOD2 receptor initiates the migration of CD4^+^ Treg cells to the lungs during IAV infection, the consequences of NOD2 triggering on CD8^+^ Treg cells remain to be investigate since CD8^+^ Tregs can also be induced during IAV infection (64).

In addition to the increased number of CD4^+^ Treg to the lung during IAV infection, MDP treatment was found to reduce Th17 cells mobilization which correlates with a decrease of neutrophil levels. We hypothesized that neutrophils infiltrate the injured lung in the early phase following infection with IAV and triggered the first wave of host defense against infection. Thereafter, Treg cells are mobilized to the lungs to attenuate excessive inflammation. NOD2 triggering may thus favor the Treg/Th17 immune balance and consequently the lung homeostasis. The precise mechanisms underlying the control of Treg/Th17 immune balance remain to be clarified.

We have previously reported that *in vivo* stimulation of NOD2 with MDP induces the emergence of circulating Ly6C^low^ patrolling monocytes (52) and also that the presence of Ly6C^low^ monocytes was required for Treg cell recruitment in a serum transfer-induced arthritis mice model (51). When performing functional depletion of blood monocytes, we showed that both Treg cell mobilization and restoration of Ly6C^low^ monocytes occurs simultaneously, a process associated with the reduction of inflammation in inflamed joints of arthritic mice. In IAV infection model, mobilization of Treg cells in lungs of mice also requires the presence of Ly6C^low^ monocytes. In this regard, these cellular populations appear to be crucial in NOD2-related signals to exert a control on IAV-induced lung inflammation and in a chronic inflammatory environment (42, 43). Furthermore, TGF-β release occurs once Ly6C^low^ monocytes are restored after monocyte depletion and when Tregs increase is detected in lungs of treated mice (51). We thus propose that treatment with MDP may promote physiological signals involving TGF-β production by mononuclear phagocytes, predominantly Ly6C^low^ monocytes, which in turn facilitate the conversion of T cells into CD4^+^ Tregs during IAV infection. Together, this study underlines the contribution of NOD2 in the control of lung inflammation during IAV infection through the mobilization of Treg cells.

## Ethics Statement

This study was carried out in accordance with the recommendations of the Guide for the Care and Use of Laboratory Animals of the Canadian Council on Animal Care (CCAC). All protocols were approved by the Committee on the Ethics of Animal Experiments of Université Laval (Approval Number: 15-109-2).

## Author Contributions

BE performed the experiments, contributed to analyze the data, and to write the manuscript. JG conceived the experiments, analyzed the data, and wrote the manuscript.

## Conflict of Interest Statement

The authors declare that the research was conducted in the absence of any commercial or financial relationships that could be construed as a potential conflict of interest.

## References

[1] Gazit R Gruda R Elboim M Arnon TI Katz G Achdout H Lethal influenza infection in the absence of the natural killer cell receptor gene Ncr1. Nat Immunol (2006) 7(5):517–23. 10.1038/ni1322 16565719

[2] La Gruta NL Kedzierska K Stambas J Doherty PC. A question of self-preservation: immunopathology in influenza virus infection. Immunol Cell Biol (2007) 85(2):85–92. 10.1038/sj.icb.7100026 17213831

[3] Treanor JJ Hayden FG Viral infections. 3rd ed In: Murray JF Nadel JA Mason R Boushey HA, editors. Textbook of Respiratory Medicine. Philadelphia, PA: W.B. Saunders (2000). p. 929–84.

[4] Takeuchi Y Nishikawa H. Roles of regulatory T cells in cancer immunity. Int Immunol (2016) 28(8):401–9. 10.1093/intimm/dxw025 27160722 PMC4986235

[5] Li Z Li D Tsun A Li B. FOXP3+ regulatory T cells and their functional regulation. Cell Mol Immunol (2015) 12(5):558–65. 10.1038/cmi.2015.10 25683611 PMC4579651

[6] Lu L Barbi J Pan F. The regulation of immune tolerance by FOXP3. Nat Rev Immunol (2017) 17(11):703–17. 10.1038/nri.2017.75 28757603 PMC5793224

[7] Arpaia N Green JA Moltedo B Arvey A Hemmers S Yuan S A distinct function of regulatory T cells in tissue protection. Cell (2015) 162(5):1078–89. 10.1016/j.cell.2015.08.021 26317471 PMC4603556

[8] Lei H Schmidt-Bleek K Dienelt A Reinke P Volk HD. Regulatory T cell-mediated anti-inflammatory effects promote successful tissue repair in both indirect and direct manners. Front Pharmacol (2015) 6:184. 10.3389/fphar.2015.00184 26388774 PMC4557110

[9] Yang WY Shao Y Lopez-Pastrana J Mai J Wang H Yang XF. Pathological conditions re-shape physiological Tregs into pathological Tregs. Burns Trauma (2015) 3(1):1–11. 10.1186/s41038-015-0001-0 26623425 PMC4662545

[10] Li MO Wan YY Sanjabi S Robertson AK Flavell RA. Transforming growth factor-beta regulation of immune responses. Annu Rev Immunol (2006) 24:99–146. 10.1146/annurev.immunol.24.021605.090737 16551245

[11] Wan YY Flavell RA. TGF-beta and regulatory T cell in immunity and autoimmunity. J Clin Immunol (2008) 28(6):647–59. 10.1007/s10875-008-9251-y 18792765 PMC2837280

[12] Fu S Zhang N Yopp AC Chen D Mao M Chen D TGF-beta induces Foxp3+ T-regulatory cells from CD4+ CD25- precursors. Am J Transplant (2004) 4(10):1614–27. 10.1111/j.1600-6143.2004.00566.x 15367216

[13] Veiga-Parga T Sehrawat S Rouse BT. Role of regulatory T cells during virus infection. Immunol Rev (2013) 255(1):182–96. 10.1111/imr.12085 23947355 PMC3748387

[14] Belkaid Y Tarbell K. Regulatory T cells in the control of host-microorganism interactions (*). Annu Rev Immunol (2009) 27:551–89. 10.1146/annurev.immunol.021908.132723 19302048

[15] Rouse BT Sarangi PP Suvas S. Regulatory T cells in virus infections. Immunol Rev (2006) 212:272–86. 10.1111/j.0105-2896.2006.00412.x 16903920

[16] Oliphant S Lines JL Hollifield ML Garvy BA. Regulatory T cells are critical for clearing influenza A virus in neonatal mice. Viral Immunol (2015) 28(10):580–9. 10.1089/vim.2015.0039 26501792 PMC4677544

[17] Moser EK Hufford MM Braciale TJ. Late engagement of CD86 after influenza virus clearance promotes recovery in a FoxP3+ regulatory T cell dependent manner. PLoS Pathog (2014) 10(8):e1004315. 10.1371/journal.ppat.1004315 25144228 PMC4140856

[18] Betts RJ Ho AW Kemeny DM Partial depletion of natural CD4(+)CD25(+) regulatory T cells with anti-CD25 antibody does not alter the course of acute influenza A virus infection. PLoS One (2011) 6(11):e27849 10.1371/journal.pone.0027849 22125630 PMC3220674

[19] Brincks EL Roberts AD Cookenham T Sell S Kohlmeier JE Blackman MA Antigen-specific memory regulatory CD4+Foxp3+ T cells control memory responses to influenza virus infection. J Immunol (2013) 190(7):3438–46. 10.4049/jimmunol.1203140 23467933 PMC3608733

[20] Perrone LA Plowden JK Garcia-Sastre A Katz JM Tumpey TM. H5N1 and 1918 pandemic influenza virus infection results in early and excessive infiltration of macrophages and neutrophils in the lungs of mice. PLoS Pathog (2008) 4(8):e1000115. 10.1371/journal.ppat.1000115 18670648 PMC2483250

[21] Sakai S Kawamata H Mantani N Kogure T Shimada Y Terasawa K Therapeutic effect of anti-macrophage inflammatory protein 2 antibody on influenza virus-induced pneumonia in mice. J Virol (2000) 74(5):2472–6. 10.1128/JVI.74.5.2472-2476.2000 10666283 PMC111734

[22] Tumpey TM Garcia-Sastre A Taubenberger JK Palese P Swayne DE Pantin-Jackwood MJ Pathogenicity of influenza viruses with genes from the 1918 pandemic virus: functional roles of alveolar macrophages and neutrophils in limiting virus replication and mortality in mice. J Virol (2005) 79(23):14933–44. 10.1128/JVI.79.23.14933-14944.2005 16282492 PMC1287592

[23] Guglani L Khader SA. Th17 cytokines in mucosal immunity and inflammation. Curr Opin HIV AIDS (2010) 5(2):120–7. 10.1097/COH.0b013e328335c2f6 20543588 PMC2892849

[24] Newcomb DC Peebles RS Jr. Th17-mediated inflammation in asthma. Curr Opin Immunol (2013) 25(6):755–60. 10.1016/j.coi.2013.08.002 24035139 PMC3855890

[25] Eisenstein EM Williams CB. The T(reg)/Th17 cell balance: a new paradigm for autoimmunity. Pediatr Res (2009) 65(5 Pt 2):26R–31R. 10.1203/PDR.0b013e31819e76c7 19218879

[26] Jamshidian A Shaygannejad V Pourazar A Zarkesh-Esfahani SH Gharagozloo M. Biased Treg/Th17 balance away from regulatory toward inflammatory phenotype in relapsed multiple sclerosis and its correlation with severity of symptoms. J Neuroimmunol (2013) 262(1–2):106–12. 10.1016/j.jneuroim.2013.06.007 23845464

[27] Yang X Li J Liu J Gao M Zhou L Lu W. Relationship of Treg/Th17 balance with HBeAg change in HBeAg-positive chronic hepatitis B patients receiving telbivudine antiviral treatment: a longitudinal observational study. Medicine (Baltimore) (2017) 96(23):e7064. 10.1097/MD.0000000000007064 28591041 PMC5466219

[28] Grimes CL Ariyananda Lde Z Melnyk JE O’Shea EK. The innate immune protein Nod2 binds directly to MDP, a bacterial cell wall fragment. J Am Chem Soc (2012) 134(33):13535–7. 10.1021/ja303883c 22857257 PMC3424850

[29] Jeong YJ Kang MJ Lee SJ Kim CH Kim JC Kim TH Nod2 and Rip2 contribute to innate immune responses in mouse neutrophils. Immunology (2014) 143(2):269–76. 10.1111/imm.12307 24766550 PMC4172142

[30] Sabbah A Chang TH Harnack R Frohlich V Tominaga K Dube PH Activation of innate immune antiviral responses by Nod2. Nat Immunol (2009) 10(10):1073–80. 10.1038/ni.1782 19701189 PMC2752345

[31] Lupfer C Thomas PG Anand PK Vogel P Milasta S Martinez J Receptor interacting protein kinase 2-mediated mitophagy regulates inflammasome activation during virus infection. Nat Immunol (2013) 14(5):480–8. 10.1038/ni.2563 23525089 PMC3631456

[32] Coulombe F Fiola S Akira S Cormier Y Gosselin J. Muramyl dipeptide induces NOD2-dependent Ly6C(high) monocyte recruitment to the lungs and protects against influenza virus infection. PLoS One (2012) 7(5):e36734. 10.1371/journal.pone.0036734 22590599 PMC3348889

[33] Rahman MK Midtling EH Svingen PA Xiong Y Bell MP Tung J The pathogen recognition receptor NOD2 regulates human FOXP3+ T cell survival. J Immunol (2010) 184(12):7247–56. 10.4049/jimmunol.0901479 20483763 PMC3886856

[34] Govorkova EA Fang HB Tan M Webster RG. Neuraminidase inhibitor-rimantadine combinations exert additive and synergistic anti-influenza virus effects in MDCK cells. Antimicrob Agents Chemother (2004) 48(12):4855–63. 10.1128/AAC.48.12.4855-4863.2004 15561867 PMC529183

[35] Sunderkotter C Nikolic T Dillon MJ Van Rooijen N Stehling M Drevets DA Subpopulations of mouse blood monocytes differ in maturation stage and inflammatory response. J Immunol (2004) 172(7):4410–7. 10.4049/jimmunol.172.7.4410 15034056

[36] Leenen PJ Radosevic K Voerman JS Salomon B van Rooijen N Klatzmann D Heterogeneity of mouse spleen dendritic cells: in vivo phagocytic activity, expression of macrophage markers, and subpopulation turnover. J Immunol (1998) 160(5):2166–73. 9498754

[37] Orimo A Gupta PB Sgroi DC Arenzana-Seisdedos F Delaunay T Naeem R Stromal fibroblasts present in invasive human breast carcinomas promote tumor growth and angiogenesis through elevated SDF-1/CXCL12 secretion. Cell (2005) 121(3):335–48. 10.1016/j.cell.2005.02.034 15882617

[38] Cambien B Richard-Fiardo P Karimdjee BF Martini V Ferrua B Pitard B CCL5 neutralization restricts cancer growth and potentiates the targeting of PDGFRbeta in colorectal carcinoma. PLoS One (2011) 6(12):e28842 10.1371/journal.pone.0028842 22205974 PMC3243667

[39] Gaudreault E Gosselin J. Leukotriene B4 induces release of antimicrobial peptides in lungs of virally infected mice. J Immunol (2008) 180(9):6211–21. 10.4049/jimmunol.180.9.6211 18424743

[40] Taubenberger JK Morens DM. The pathology of influenza virus infections. Annu Rev Pathol (2008) 3:499–522. 10.1146/annurev.pathmechdis.3.121806.154316 18039138 PMC2504709

[41] Pelletier M Maggi L Micheletti A Lazzeri E Tamassia N Costantini C Evidence for a cross-talk between human neutrophils and Th17 cells. Blood (2010) 115(2):335–43. 10.1182/blood-2009-04-216085 19890092

[42] Wojkowska DW Szpakowski P Ksiazek-Winiarek D Leszczynski M Glabinski A. Interactions between neutrophils, Th17 cells, and chemokines during the initiation of experimental model of multiple sclerosis. Mediators Inflamm (2014) 2014:590409. 10.1155/2014/590409 24692851 PMC3945772

[43] Stoppelenburg AJ Salimi V Hennus M Plantinga M Huis in ’t Veld R Walk J Local IL-17A potentiates early neutrophil recruitment to the respiratory tract during severe RSV infection. PLoS One (2013) 8(10):e78461. 10.1371/journal.pone.0078461 24194936 PMC3806820

[44] Brackett CM Muhitch JB Evans SS Gollnick SO. IL-17 promotes neutrophil entry into tumor-draining lymph nodes following induction of sterile inflammation. J Immunol (2013) 191(8):4348–57. 10.4049/jimmunol.1103621 24026079 PMC3795982

[45] Katayama M Ohmura K Yukawa N Terao C Hashimoto M Yoshifuji H Neutrophils are essential as a source of IL-17 in the effector phase of arthritis. PLoS One (2013) 8(5):e62231. 10.1371/journal.pone.0062231 23671588 PMC3646022

[46] Witowski J Pawlaczyk K Breborowicz A Scheuren A Kuzlan-Pawlaczyk M Wisniewska J IL-17 stimulates intraperitoneal neutrophil infiltration through the release of GRO alpha chemokine from mesothelial cells. J Immunol (2000) 165(10):5814–21. 10.4049/jimmunol.165.10.5814 11067941

[47] Wei S Kryczek I Edwards RP Zou L Szeliga W Banerjee M Interleukin-2 administration alters the CD4+FOXP3+ T-cell pool and tumor trafficking in patients with ovarian carcinoma. Cancer Res (2007) 67(15):7487–94. 10.1158/0008-5472.CAN-07-0565 17671219

[48] Wysocki CA Jiang Q Panoskaltsis-Mortari A Taylor PA McKinnon KP Su L Critical role for CCR5 in the function of donor CD4+CD25+ regulatory T cells during acute graft-versus-host disease. Blood (2005) 106(9):3300–7. 10.1182/blood-2005-04-1632 16002422 PMC1895335

[49] Levings MK Bacchetta R Schulz U Roncarolo MG. The role of IL-10 and TGF-beta in the differentiation and effector function of T regulatory cells. Int Arch Allergy Immunol (2002) 129(4):263–76. 10.1159/000067596 12483031

[50] Schmitt EG Haribhai D Williams JB Aggarwal P Jia S Charbonnier LM IL-10 produced by induced regulatory T cells (iTregs) controls colitis and pathogenic ex-iTregs during immunotherapy. J Immunol (2012) 189(12):5638–48. 10.4049/jimmunol.1200936 23125413 PMC3537488

[51] Brunet A LeBel M Egarnes B Paquet-Bouchard C Lessard AJ Brown JP NR4A1-dependent Ly6Clow monocytes contribute to reducing joint inflammation in arthritic mice through Treg cells. Eur J Immunol (2016) 46(12):2789–800. 10.1002/eji.201646406 27600773

[52] Lessard AJ LeBel M Egarnes B Prefontaine P Theriault P Droit A Triggering of NOD2 receptor converts inflammatory Ly6Chigh into Ly6Clow monocytes with patrolling properties. Cell Rep (2017) 20(8):1830–43. 10.1016/j.celrep.2017.08.009 28834747

[53] Horwitz DA Zheng SG Gray JD. The role of the combination of IL-2 and TGF-beta or IL-10 in the generation and function of CD4+ CD25+ and CD8+ regulatory T cell subsets. J Leukoc Biol (2003) 74(4):471–8. 10.1189/jlb.0503228 14519757 PMC7166542

[54] Rutz S Janke M Kassner N Hohnstein T Krueger M Scheffold A. Notch regulates IL-10 production by T helper 1 cells. Proc Natl Acad Sci U S A (2008) 105(9):3497–502. 10.1073/pnas.0712102105 18292228 PMC2265185

[55] Chaudhry A Samstein RM Treuting P Liang Y Pils MC Heinrich JM Interleukin-10 signaling in regulatory T cells is required for suppression of Th17 cell-mediated inflammation. Immunity (2011) 34(4):566–78. 10.1016/j.immuni.2011.03.018 21511185 PMC3088485

[56] Chen W Jin W Hardegen N Lei KJ Li L Marinos N Conversion of peripheral CD4+CD25- naive T cells to CD4+CD25+ regulatory T cells by TGF-beta induction of transcription factor Foxp3. J Exp Med (2003) 198(12):1875–86. 10.1084/jem.20030152 14676299 PMC2194145

[57] Blankenhaus B Reitz M Brenz Y Eschbach ML Hartmann W Haben I Foxp3(+) regulatory T cells delay expulsion of intestinal nematodes by suppression of IL-9-driven mast cell activation in BALB/c but not in C57BL/6 mice. PLoS Pathog (2014) 10(2):e1003913 10.1371/journal.ppat.1003913 24516385 PMC3916398

[58] Selvaraj RK Geiger TL A kinetic and dynamic analysis of Foxp3 induced in T cells by TGF-beta. J Immunol (2007) 178(12):7667–77. 10.4049/jimmunol.178.12.7667 17548603

[59] Belkaid Y Rouse BT. Natural regulatory T cells in infectious disease. Nat Immunol (2005) 6(4):353–60. 10.1038/ni1181 15785761

[60] Losikoff PT Self AA Gregory SH. Dendritic cells, regulatory T cells and the pathogenesis of chronic hepatitis C. Virulence (2012) 3(7):610–20. 10.4161/viru.21823 23076334 PMC3545943

[61] Cui W Liu Y Weinstein JS Craft J Kaech SM. An interleukin-21-interleukin-10-STAT3 pathway is critical for functional maturation of memory CD8+ T cells. Immunity (2011) 35(5):792–805. 10.1016/j.immuni.2011.09.017 22118527 PMC3431922

[62] Foulds KE Rotte MJ Seder RA. IL-10 is required for optimal CD8 T cell memory following *Listeria monocytogenes* infection. J Immunol (2006) 177(4):2565–74. 10.4049/jimmunol.177.4.2565 16888018

[63] Laidlaw BJ Cui W Amezquita RA Gray SM Guan T Lu Y Production of IL-10 by CD4(+) regulatory T cells during the resolution of infection promotes the maturation of memory CD8(+) T cells. Nat Immunol (2015) 16(8):871–9. 10.1038/ni.3224 26147684 PMC4713030

[64] Zou Q Wu B Xue J Fan X Feng C Geng S CD8+ Treg cells suppress CD8+ T cell-responses by IL-10-dependent mechanism during H5N1 influenza virus infection. Eur J Immunol (2014) 44(1):103–14. 10.1002/eji.201343583 24114149 PMC4165276

